# DNA methylation of *ACADS* promotes immunogenic cell death in hepatocellular carcinoma

**DOI:** 10.1186/s13578-024-01334-1

**Published:** 2025-01-12

**Authors:** Ze Qian, Yifan Jiang, Yacong Wang, Yu Li, Lin Zhang, Xiaofeng Xu, Diyu Chen

**Affiliations:** 1 Division of Hepatobiliary and Pancreatic Surgery, Department of Surgery, First Affiliated Hospital, School of Medicine, Zhejiang University, Hangzhou, 310003 Zhejiang China; 2 Department of Gerontology, First Affiliated Hospital, Zhejiang University School of Medicine, Hangzhou, 310003 Zhejiang China; 3 Department of Pharmacology, Shaoxing People’s Hospital, Shaoxing, 312035 Zhejiang Province China

**Keywords:** ACADS, HCC, DNA methylation, ICD, Tumour microenvironment

## Abstract

**Background:**

Altered metabolism has become an important characteristic of cancer, and acyl-CoA dehydrogenase short-chain (ACADS), a regulator of lipid synthesis, is involved in carcinogenesis-associated metabolic pathways. DNA methylation is an important mechanism for silencing ACADS in various malignancies. However, the specific role of ACADS in hepatocellular carcinoma (HCC) pathogenesis remains poorly understood.

**Methods and results:**

Using RNA sequencing data from different tumours in The Cancer Genome Atlas database, we observed that *ACADS* was downregulated and hypermethylated in HCC. Three potential CpG island sites (cg01535453, cg08618068, and cg10174836) were identified in the *ACADS* promoter. Through in vivo and in vitro experiments, we confirmed that cg08618068 was methylated in HCC. We defined this site as ACADS methylation site-2 (ACADS MS-2). Methylation of ACADS MS-2 was associated with worse survival, and mutation of MS-2 increased *ACADS* mRNA levels in five HCC cell lines. Sustained overexpression of *ACADS* not only suppressed the proliferation, migration, and invasion of HCC cells but also promoted immunogenic cell death (ICD) via the upregulation of calreticulin. Subsequently, we established a specific nomogram based on *ACADS* methylation levels to evaluate the 3- and 5-year overall survival rates of patients with HCC who underwent surgical resection.

**Conclusions:**

Our work clarified that ACADS acts as a putative tumour suppressor in HCC and confirmed that a nomogram including *ACADS* methylation had good predictive performance in HCC. We also discovered a correlation between ACADS and ICD, suggesting that ACADS is an essential target for immunotherapy in HCC.

**Graphical Abstract:**

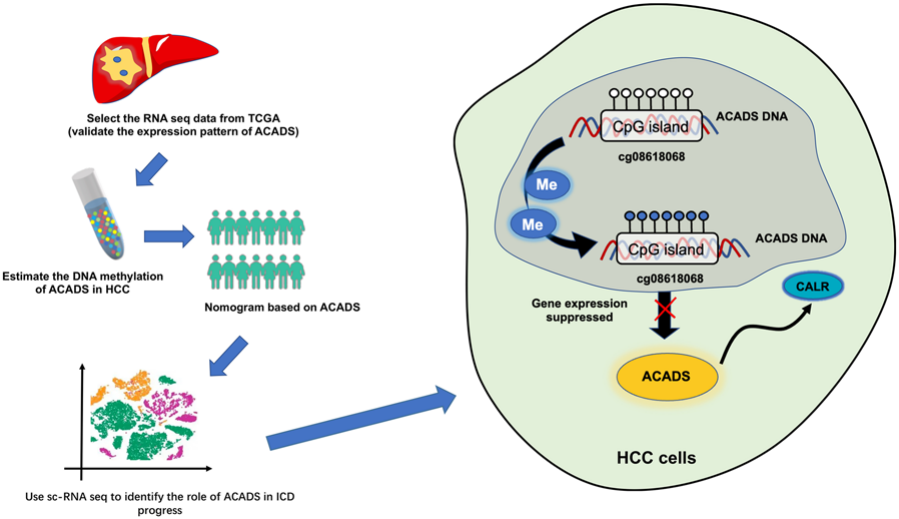

**Supplementary Information:**

The online version contains supplementary material available at 10.1186/s13578-024-01334-1.

## Background

Owing to the increasing incidence of hepatocellular carcinoma (HCC) in recent decades, it is now one of the highest causes of cancer-related death worldwide [1–3]. Surgery is the primary method of HCC treatment [2, 4, 5]. However, the available therapeutic options for patients with advanced disease remain extremely limited [6, 7]. Therefore, investigation of the regulatory mechanisms underlying the aetiology and pathogenesis of HCC is urgently required.

Accumulating evidence indicates that epigenetic variants are increasingly important in promoting carcinogenesis [8, 9]. As a special form of epigenetic modification, DNA methylation is an attractive possibility to stimulate the oncogenic transformation of HCC, especially when methylation or hypermethylation occurs at histones near the DNA segments of tumour suppressor genes (TSGs) [9–11]. Therefore, potential DNA methylation-mediated candidates involved in hepatocellular carcinogenesis need to be identified.

Tumour cell death can be immunogenic or non-immunogenic based on divergent initiating stimuli [12]. Immunogenic cell death (ICD) involves changes in the composition of cell surface markers as well as the release of soluble mediators occurring in a defined temporal sequence. These signals operate on a series of receptors expressed by dendritic cells (DCs) to stimulate the presentation of tumour antigens to T cells, blocking aberrant tumour expansion and determining the long-term success of anticancer therapies [12, 13]. Additionally, ICD can be influenced by epigenetic modifications [14]. However, it remains unclear whether DNA methylation is essential for driving ICD in HCC.

Altered metabolism in malignancies has become an important characteristic of cancer [15–17]. Lipids are important metabolic products that play major roles in HCC progression [18, 19]. Several regulators of lipid synthesis have gained considerable attention because of their complex roles in tumour development. Among these, acyl-CoA dehydrogenase (ACAD) genes encode a family of enzymes that are responsible for fatty acid metabolism [20]. ACAD proteins comprise a pan-taxonomic protein family [21, 22]. Distinguished by their substrate specificity, the ACAD enzymes could be divided into acyl-CoA dehydrogenase short-chain (ACADS), acyl-CoA dehydrogenase medium chain, acyl-CoA dehydrogenase long chain, ACADV, and ACADV2 [20, 23]. ACADs are key enzymes involved in carcinogenesis-associated metabolic pathways, and many studies have revealed that ACAD could be a novel biomarker for cancer. However, the role of ACADS in HCC progression remains poorly understood.

## Methods

### Cell culture and data sources

The HCC cell lines used in this study were obtained from the Cell Bank of the Shanghai Institutes for Biological Sciences, Chinese Academy of Sciences, Shanghai, China. Minimum essential Media (MEM) containing 10% foetal bovine serum was used to maintain these cells. Hepa1-6 cells were cultured in RPMI supplemented with 10% FBS, 100 U/mL penicillin, and 100 μg/mL streptomycin. All the HCC cell lines were cultured in a humidified incubator at 37 °C and 5% CO_2_.

The extensive bioinformatics analyses undertaken in this study utilized data exclusively sourced from the MEXPRESS database (https://mexpress.be/) and The Cancer Genome Atlas (TCGA) database (https://cancergenome.nih.gov/).

### RNA isolation and quantitative real-time PCR (qRT-PCR)

Total RNA was isolated using the TRIzol reagent (TaKaRa, China). A cDNA library was synthesized with the PrimeScript RT Reagent Kit (TaKaRa, China). Quantitative real-time PCR analyses were performed using the SYBR Green PCR Kit (TaKaRa, Hangzhou, China) on an ABI 7500 FAST Real-Time PCR system (Applied Biosystems, America). Relative mRNA expression levels were quantified using the 2ΔΔCt method and normalized against GAPDH, which served as the internal reference gene.

### Establishment of ACADS-overexpressing cell lines

Two ACADS-low cell lines (Huh-7 and HCC-LM3) were used to perform ACADS overexpression assays. We used a lentiviral vector containing ACADS (Ribort, Hangzhou, China). A non-specific lentiviral vector was used as a negative control (NC). We transfected the NC or ACADS overexpression vectors into tumour cell lines in the presence of polybrene (8 µg/mL) for 24 h. To select the transfected cells, blasticidin (10 µg/mL) was added into the medium for 72 h, and surviving cells were seeded in six-well plates. RT-qPCR and western blotting were used to evaluate the efficiency of exogenous ACADS overexpression in HCC cells.

### Proliferation and colony formation assays

For the Cell Counting Kit (CCK)−8 assay, we seeded HCC cells in 96-well plates at a density of 1200–1500 cells/well. The CCK-8 assay was purchased from Dojindo (Kyushu, Japan). CCK-8 was added to wells at 24 h, 48 h, or 72 h, and the absorbance was measured at 450 nm. For the colony formation assay, cells were seeded into six-well plates at a density of 1000 cells per well. The cells were cultured at 37 ℃ for 2 weeks. Then, cells were fixed with 100% methanol and stained with 0.1% crystal violet. Next, the colonies were counted using Image-Pro Plus 5.0 software (Media Cybernetics).

### EdU assay

HCC cells (2 × 10^5^) were seeded in 24-well plates and incubated for 24 h. The EdU assays were conducted using a 5-ethynyl-2′-deoxyuridine (EdU) cell proliferation assay kit (UElandy, Hangzhou, China). A 0.1 mL aliquot of 50 µM EdU was added to each well containing 500 µL of medium and the cells were incubated for an additional 2 h. Subsequently, the cells were fixed with 4% paraformaldehyde in PBS at room temperature for 30 min, followed by incubation with Apollo staining solution and Hoechst 33342 for 30 min. Fluorescence microscopy was utilized to examine the stained cells.

### Migration and invasion assays

For the cell invasion assay, 35 μL of a mixed liquid solution containing Matrigel (BD Biosciences, Washington, USA) and MEM in a 1:7 ratio was added to a Transwell insert, which was incubated in a humidified incubator at 37 ℃ for 3 h. Then, 3 × 10^4^ or 5 × 10^4^ HCC cells in serum-free medium were added to the upper chamber. Medium containing 10% foetal bovine serum was added to the lower chamber. The cells were cultured at 37 ℃ and 5% CO_2_ for 48 h for the cell migration assay and 72 h for the cell invasion assay. A Wright-Giemsa staining kit was used to stain the cells on the surface of the membrane.

### Wound healing assay

In a 24-well plate, HCCLM3 and Huh-7 cells were seeded at a density of 5 × 10^5^ cells per well. Upon reaching 90% confluence, a sterile 200 μL pipette tip was used to create a scratch in the monolayer. Cells were then washed thrice with phosphate-buffered saline (PBS) to remove detached cells, followed by the addition of serum-free medium. The cells were cultured in an incubator. Microscopic images were captured at 0 and 72 h post-scratch.

### Flow cytometry assay

Apoptosis in hepatocellular carcinoma cells was detected using flow cytometry. Cells were harvested by trypsinization and PBS washing 48 h post-stable transfection. Subsequently, they were fixed in 70% cold ethanol at 4 ℃ for 24 h. After centrifugation and further washes, cells were incubated with 500 μL of propidium iodide (PI) staining solution (50 μg/mL) shielded from light, at 37 ℃ for 30 min. Following staining, cells were analyzed with a flow cytometer, and apoptosis levels were determined based on the apoptosis detection kit's instructions.

### DNA methylation immunoprecipitation analysis (Me-DIP)

In our study, we processed 20 liver cancer specimens along with paired normal liver tissues from the same individuals, which were procured from our institution. These samples underwent cellular dissociation and DNA isolation. We then utilized a Me-DIP kit (Biocytogen, Hangzhou, China) to assess the levels of DNA methylation modifications. Furthermore, to extend our investigation into the DNA methylation patterns in liver cancer, we incorporated additional MeDIP data for the ACADS gene, derived from liver carcinoma cases, as well as corresponding clinical information, obtained from The Cancer Genome Atlas (TCGA) database (https://cancergenome.nih.gov/).

### Bisulfite sequencing PCR (BSP)

In the investigation, we initially extracted genomic DNA from neoplastic cell lines utilizing a commercial DNA isolation kit. This was succeeded by treatment with the DNA Methylation-Gold™ kit. To corroborate the bioinformatics outcomes derived from tissue specimens, we conducted Bisulfite Sequencing PCR (BSP). Throughout the sequencing protocol, amplified DNA samples underwent PCR and subsequent fragmentation. The PCR reaction was conducted in a 50 μL mixture, comprising 2 μL of template DNA, 10 μL of 5× GC-rich buffer, 0.2 μL of Taq polymerase (5 U/μL), 1μL of dNTPs (10 mM), and 1μL of each primer, with the volume completed to 50 μL using ultra-pure water. The amplification conditions were as follows: an initial denaturation at 95 °C for 3 min, followed by 40 cycles of denaturation at 94 °C for 30 s, annealing at 54 °C for 30 s, and extension at 72 °C for 1 min, concluding with a final extension at 72 °C for 7 min. The PyroMark Q96 system (Qiagen, Germany) was subsequently employed for sample precipitation, resuspension, and genotyping analysis.

### Immunofluorescent staining assays

Tumor specimens from two groups of mice were procured for immunohistochemical fluorescence analysis (referred to as the ACADS-OE group and the NC group, respectively). The specimens underwent overnight incubation with the ACADS antibody at 4 °C. Subsequent to meticulous rinsing, the tissues were incubated with a specified horseradish peroxidase (HRP)-conjugated, fluorescent secondary antibody. The expression of ACADS was visualized using 3,3ʹ-diaminobenzidine tetrahydrochloride (DAB).

### Dual-luciferase reporter gene assay

Employing the pGL3 vector, which spans ~ 5 kb, this construct harbors the luciferase (Luc) gene under the control of a specific promoter segment inserted upstream. Following stable co-transfection, hepatocellular carcinoma cell lines were categorized into two primary groups: the cg08618068-wild type-luc (WT-luc) and the cg08618068-mutant type-luc (MT-luc) groups. Each group was subdivided, with one set receiving saline and the other treated with the DNA methylation stabilizer SS1. Luciferase activity was subsequently quantified, facilitating a comparative analysis among the subgroups.

### 5-azacytidine-deoxycytidine treatment

HCC cell lines were treated with 10 mM 5-azacitidine-deoxycytidine (Sigma-Aldrich) for 96 h. Total RNA was extracted from each sample to estimate *ACADS* expression.

### In vivo tumorigenicity assays

The in vivo experiments in this study were performed according to the rules and regulations established by the National Institutes of Health (Guide for the Care and Use of Laboratory Animals, 2011). Nude mice were used for the xenogeneic subcutaneous tumour model. NC or ACADS-OE Huh-7 cells were resuspended in 100 μL PBS and injected into the left flank of nude mice (5 × 10^6^ cells/mouse). All the mice were sacrificed on the 30th day. For the orthotopic tumour model, C57BL/6 mice (6–10 weeks) were housed at standard room temperature with food and water provided ad libitum. A 25-μL mixture of PBS and Basement Membrane Matrix (with the ratio of 1:1354248, Corning, Matrigel) containing 5 × 10^4^ Hepa1-6 cells was injected into the left liver lobe of male C57BL/6 mice to establish the orthotopic HCC model. Tumour volumes were calculated using the modified ellipsoid formula ((length × width^2^)/2).

### Single-cell sequencing analysis

Mice bearing tumors with ACADS protein overexpression, alongside negative controls, were gathered into the ACADS-OE group and the NC-OE group, respectively. The initial step involved excising peripheral fat, connective tissue, and necrotic regions from the samples. Following this, the samples were rinsed with chilled phosphate-buffered saline (PBS), preserved in a PBS-based solution, and promptly conveyed on ice to the dissociation lab. It was crucial to maintain a minimum tissue sample weight of 200 mg to ensure the collection of intact, viable target cells. Subsequent to cell extraction, single-cell sequencing analysis was conducted utilizing the 10× Genomics platform.

### Data processing of differentially expressed genes (DEGs)

We utilized the GEO2R online analysis tool from NCBI (available at https://www.ncbi.nlm.nih.gov/geo/geo2r/, accessed on 1 May 2023) to identify DEGs between tumor and adjacent normal tissues. The threshold for significance was set at an adjusted p-value < 0.05 combined with an absolute log fold change (|logFC|) of ≥ 2.0. Statistical analyses were conducted for each dataset independently. Venn diagrams were generated using the web-based tool at https://bioinformatics.psb.ugent.be/webtools/Venn/, which facilitated the comparison of DEGs across datasets.

### GO and KEGG pathway analysis of DEGs

We performed Gene Ontology (GO) annotation and Kyoto Encyclopedia of Genes and Genomes (KEGG) pathway enrichment analyses for the DEGs using the DAVID tools (https://david.ncifcrf.gov/, accessed on 1 May 2023). Criteria for statistical significance were set at p < 0.01 and a minimum gene count of ≥ 10.

### Statistical analysis

Demographic, clinicopathological, and therapeutic characteristics are described as summative statistics and expressed as percentages or median values. They were obtained using a mature methodology. Time to death and censored data were evaluated using the Kaplan–Meier method, and differences were compared using the log-rank test. All statistical tests were two-tailed.

The primary goal of nomogram construction was to design a robust model with fewer than 10 predictive parameters. All clinical variables were considered in the analysis of patient data obtained from the TCGA database. In terms of tumour staging data, we considered both AJCC staging and the individual variables included in AJCC staging. The continuous predictor used a cubic spline transform to maximise the Wald-χ^2^ statistic. Within-hospital clustering of patients was also considered. Based on the Akaike information criterion, backward stepwise selection was performed, and the factors were included in the multivariate Cox proportional hazards regression model. Hazard ratios and 95% CIs were evaluated. The Kaplan–Meier curve of the predicted quartile was drawn using the discriminant represented by Harrell’s C index, and a calibration curve was drawn using bootstrap samples to further illustrate the performance of the model. Statistical analyses were performed using SAS, version 9.3 (SAS Institute Inc.) and R (version 2.15.3) (http://www.r-project.org/) software packages.

## Results

### Decreased ACADS predicts poor prognosis in HCC

**Fig. 1:**
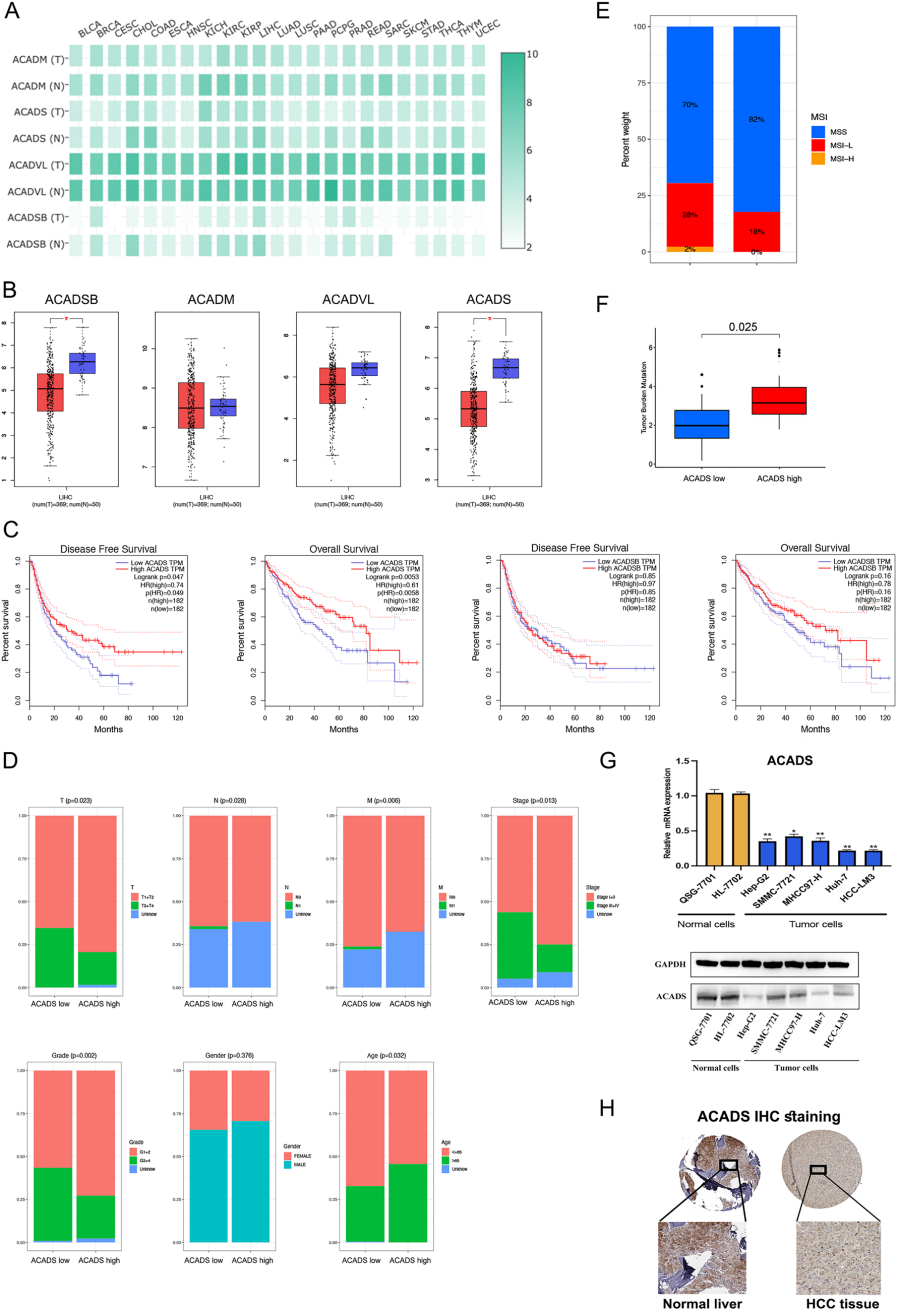
ACADS is downregulated in HCC tissues. **A** Pan-cancer analysis of ACAD family member (including ACADS, ACADM, ACADSB, and ACADVL) expression in tumour and non-tumour tissues. All data were obtained from the TCGA database. **B** Box plots showing ACADSB and ACADS expression in HCC and normal liver samples. **C** Kaplan–Meier curve showing OS and TFS in HCC based on ACADS/ACADSB expression. **D** The relationship between clinicopathological features and ACADS expression. **E**, **F** ACADS expression influences MSS status and TMB distribution in patients with HCC. **G** Using RT-qPCR and Western blot, we examined the mRNA and protein levels of ACADS in normal liver cells (QSG-7701 and HL-7702) and HCC cells (Hep-G2, SMMC-7721, MHCC97-H, Huh-7, and HCC-LM3). **H** The protein level of ACADS was reduced in tumour samples using IHC (*P < 0.05, **P < 0.01)

Several studies have suggested that the ACAD family could potentially be novel therapeutic targets for tumourigenesis because of their roles in lipid metabolism. Therefore, verifying the specific role of ACAD family members in HCC development is necessary [21, 22]. We initially validated the expression of the ACAD family members in different cancer types using The Cancer Genome Atlas (TCGA) database. Most of these genes were decreased in cancer (Fig. 1A). Moreover, as shown in the boxplots in Fig. 1B, *ACADS* and acyl-CoA dehydrogenase short/branched chain were significantly differentially expressed between HCC and adjacent normal tissues. The results of Kaplan–Meier analysis also revealed that HCC patients with high *ACADS* expression had better overall survival (OS) and tumour-free survival (TFS) than those with low *ACADS* expression (Fig. 1C and Fig. S1A). We next divided HCC patients from TCGA into high and low ACADS expression groups and examined the clinicopathological differences. The low ACADS group had a higher TNM stage and pathological grade than the high ACADS group (Fig. 1D and Fig. S1B). We also found a reduced tumour mutation burden and frequent microsatellite instability in the low ACADS group (Fig. 1E, F). Additionally, we separately evaluated the mRNA and protein levels of ACADS in cell and tumour resection samples using RT-qPCR, Western blot and immunohistochemistry (IHC) staining (Fig. 1G, H and Fig. S1C). These results confirmed that ACADS expression is decreased in HCC cell lines and specimens. Based on these findings, we hypothesized that ACADS could act as a TSG in HCC.

### ACADS has a tumour-suppressive effect in HCC

**Fig. 2:**
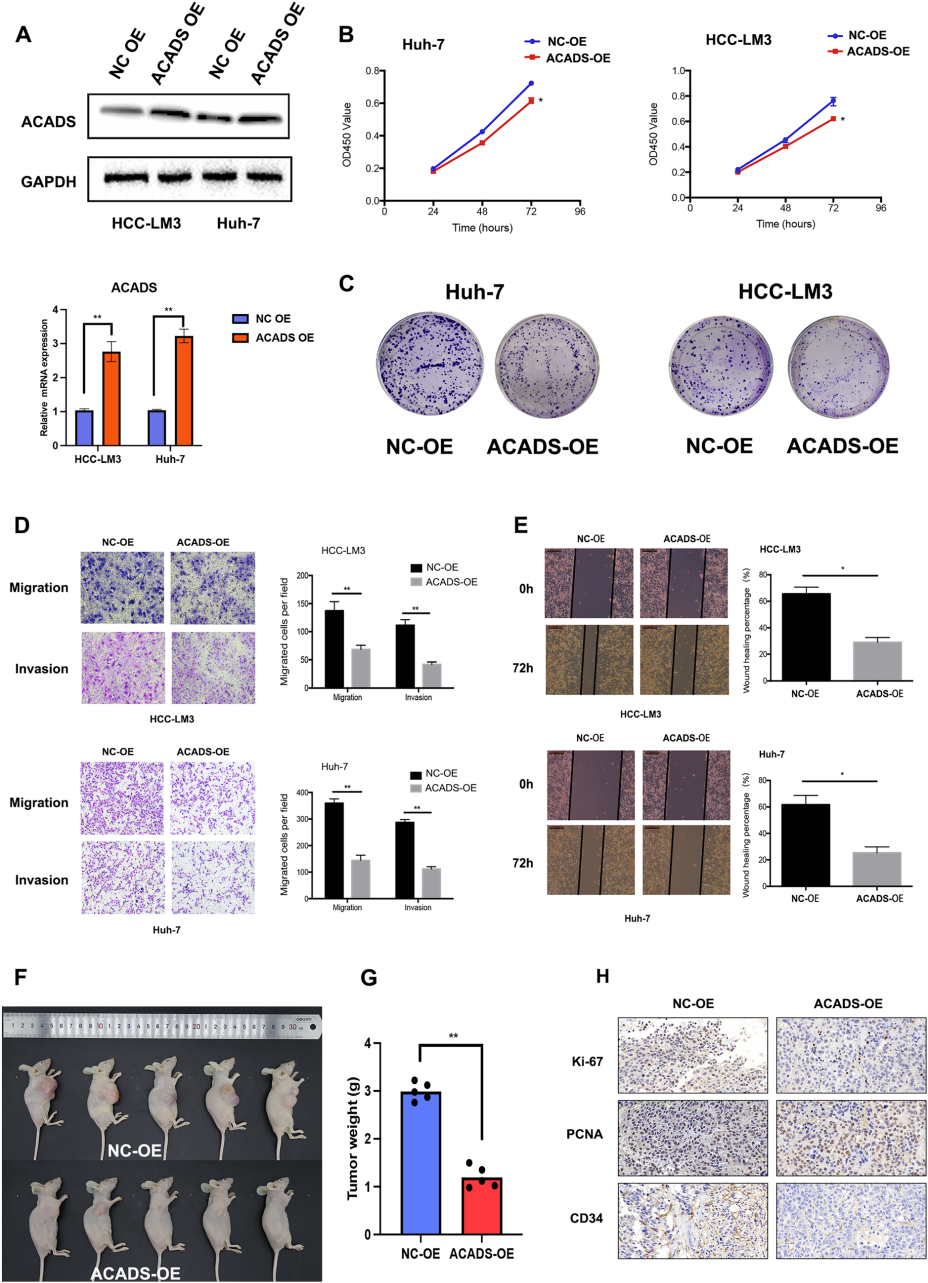
ACADS acts as a tumour suppressor in HCC. **A** After transfection of the ACADS-OE plasmid, the protein and mRNA levels of ACADS increased in Huh-7 and HCC-LM3 cells. **B** The CCK-8 assay was performed to assess the proliferation of HCC cells after ACADS overexpression at each timepoint (24 h, 48 h, and 72 h). **C** ACADS-OE cells formed more colonies than NC cells. **D** The effects of ACADS overexpression on cell migration and invasion were determined using Transwell assays. Representative micrographs of the Transwell assays are presented. **E** Similar effects were observed with respect to cell motility in the wound-healing assay. **F** Overexpression of ACADS suppressed tumour expansion in a xenograft model of HCC. **G** Weights of the tumour specimens from each group. **H** Expression of Ki-67, PCNA, and CD34 in tumours from different groups using IHC (*P < 0.05, **P < 0.01)

To understand the contribution of ACADS in HCC, we chose two mildly-expressed ACADS HCC cell lines (Huh-7 and HCC-LM3) to assess the effect of function assays. First, we used a lentiviral vector to overexpress ACADS. As shown in Fig. 2A, ACADS levels were significantly higher in the stable overexpression (OE) cells than in the NC cells (*P* < 0.01). CCK-8 assays were conducted to compare the cell growth in NC and ACADS-OE cells. ACADS-OE cells had less proliferation, indicating that ACADS expression repressed the proliferation of HCC cells (Fig. 2B, P < 0.05). Similarly, ACADS-OE cells grew fewer cell colonies than NC cells (Fig. 2C). Transwell and wound healing assays showed that ACADS OE impaired HCC cell migration and invasion (Fig. 2D–E, *P < 0.05, **P < 0.01). We also observed that ACADS overexpression could induce the raised expression of E-Cadherin. Finally, protein levels of N-Cadherin and Snail both decreased, followed by the up-regulation of ACADS in HCC cells (Fig. S1D). Subsequently, we used the NC and ACADS-OE HCC cells to establish a xenogeneic subcutaneous HCC model. ACADS OE strongly suppressed the aberrant expansion of HCC cells (Fig. 2F, G, P < 0.01). We further performed IHC on these tumours, and the ACADS-OE tumours had decreased staining intensities of Ki-67, PCNA, and CD34 when compared to NC tumours (Fig. 2H). Overall, these results indicate that ACADS reduces the migration, invasion, and proliferation abilities of HCC cells.

### DNA methylation is used to silence *ACADS* in HCC

Many studies have revealed that DNA can be used to maintain genomic suppression in cancer. Pan-cancer DNA methylation bioinformatics analysis was employed to explore the DNA methylation patterns of *ACADS*. By analyzing data from the MEXPRESS database, it has been found that the ACADS DNA methylation levels in most cancers are higher than in normal tissues (Table 1). We treated HCC cells with 5-Aza-deoxycytidine, a DNA methylation inhibitor, and *ACADS* mRNA expression was increased after treatment, suggesting that *ACADS* is regulated by DNA methylation (Fig. S2A).

**Table 1:** The DNA methylation level of ACADS in different tumor types (from MEXPRESS database)

Cancer	Cancer number	Normal number	Cancer Q2	Normal Q2	P value
LAML	140	1	0.0345	0	NA
ACC	80	1	0.0375	0	NA
BLCA	416	21	0.0366	0.0422	7.29E−01
LGG	534	1	0.4576	0	NA
BRCA	794	96	0.0559	0.0505	1.10E−05
CESC	309	3	0.0384	0.0374	7.07E−01
CHOL	36	9	0.0443	0.0394	9.28E−02
COAD	309	38	0.0382	0.0364	3.45E−02
ESCA	186	16	0.0365	0.0346	5.32E−01
GBM	153	2	0.0497	0.0612	8.15E−01
HNSC	530	50	0.0449	0.0458	1.11E−02
KICH	66	1	0.0382	0	NA
KIRC	323	160	0.0353	0.0309	8.93E−09
KIRP	276	45	0.0344	0.0358	4.03E−01
LIHC	380	50	0.0395	0.0379	2.58E−02
LUAD	471	32	0.0672	0.0639	1.34E−01
LUSC	370	42	0.0381	0.0314	3.61E−02
DLBC	48	1	0.0386	0	NA
MESO	87	1	0.038	0	NA
OV	10	1	0.0367	0	NA
PAAD	185	10	0.0379	0.0311	4.06E−01
PCPG	184	3	0.0609	0.0342	3.22E−01
PRAD	503	50	0.0456	0.0409	2.28E−02
READ	99	7	0.0359	0.043	1.81E−02
SARC	265	4	0.0376	0.0287	4.52E−01
SKCM	473	2	0.0386	0.0405	6.50E−01
STAD	395	2	0.037	0.0712	7.03E−03
TGCT	156	1	0.0392	0	NA
THYM	124	2	0.0513	0.045	6.04E−01
THCA	515	56	0.0397	0.0416	7.32E−01
UCS	57	1	0.039	0	NA
UCEC	436	46	0.0396	0.0403	4.80E−01
UVM	80	1	0.0325	0	NA

**Fig. 3:**
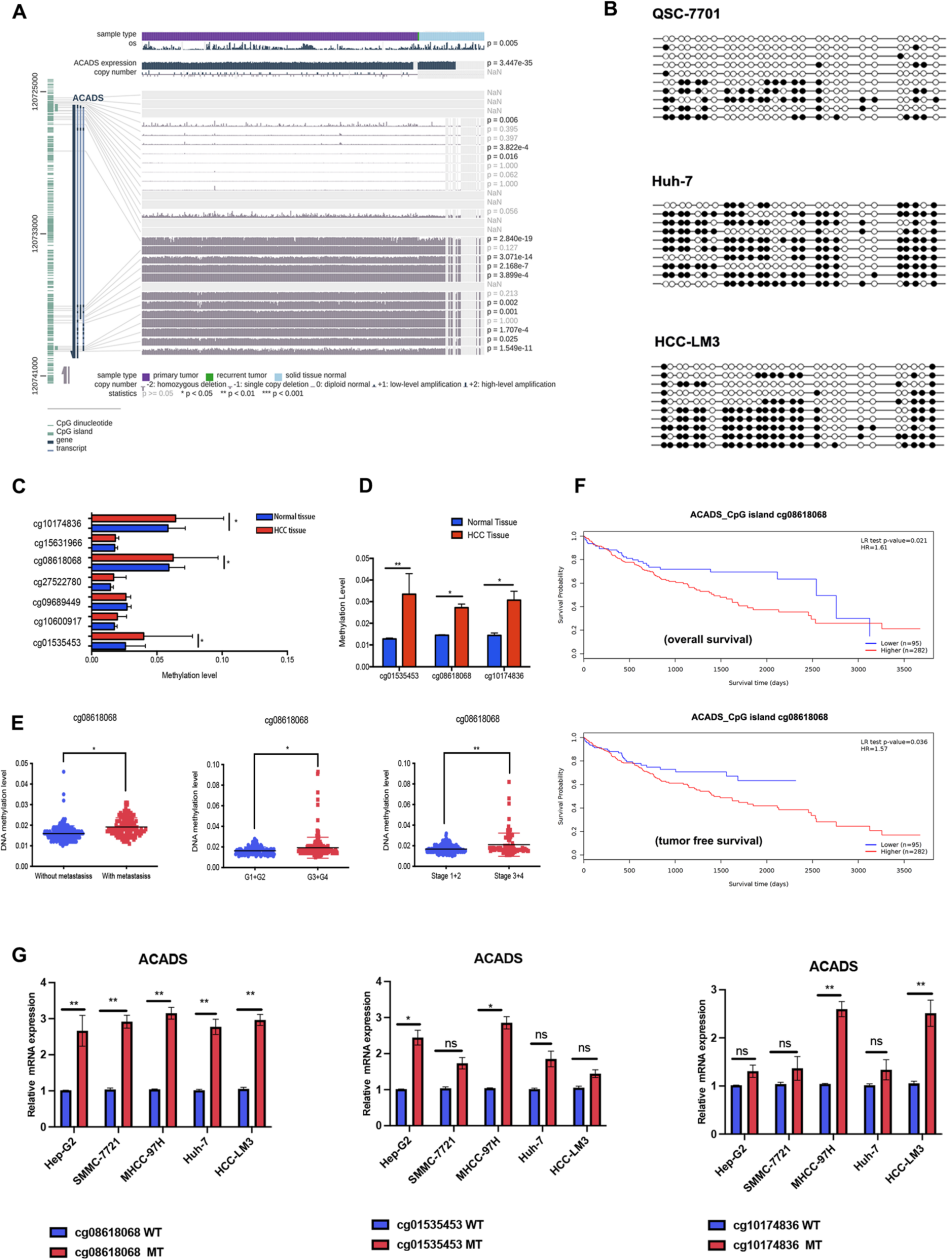
Identifying the key CpG-island site in the *ACADS* promoter. **A** DNA methylation in HCC and normal liver tissues from the MEXPRESS database. **B** BSP was used to assess *ACADS* promoter methylation in HCC cells (Huh-7 and HCC-LM3) compared to noncancerous cells (QSC-7701). White and black circles represent nonmethylated and methylated CpG sites, respectively; rows represent individual clones; and columns represent individual CpG sites within the *ACADS* promoter region. **C**, **D** Me-DIP data obtained from TCGA (**C**) and our centre (**D**) identified three potential CpG island sites: ACADS MS-1 (cg10174836), ACADS MS-2 (cg08618068), and ACADS MS-3 (cg01535453). **E** Relationship between metastasis, TNM stage, pathological grade, and ACADS MS-2 methylation levels in HCC tissues. **F** Kaplan–Meier curves of OS and TFS based on ACADS MS-2 methylation. **G** Following mutation of ACADS MS-2, ACADS mRNA expression was decreased in all HCC cell lines (*P < 0.05, **P < 0.01)

CpG islands play an important role in silencing TSGs through hypermethylation. Therefore, we examined methylated DNA immunoprecipitation (Me-DIP) sequencing data from TCGA for subsequent analysis. The methylation levels at several CpG island sites in the *ACADS* promoter were enhanced in HCC compared to those in adjacent normal tissue (Fig. 3A). Bisulfite sequencing PCR (BSP) was used to confirm the DNA methylation status of the *ACADS* promoter in vitro. The results indicated that CpG islands in the *ACADS* promoter were hypermethylated in HCC cells compared to non-cancerous cells (Fig. 3B). These data suggest that ACADS may be transcriptionally repressed by DNA methylation in HCC.

### Methylation of the CpG island cg08618068 (ACADS MS-2) decreases *ACADS* levels in HCC

To determine the specific CpG island site in the *ACADS* promoter that regulates its expression in cancer, we comprehensively analysed Me-DIP sequences from TCGA (Fig. 3C) and our centre (Fig. 3D). In both datasets, three CpG island sites (cg01535453, cg08618068, and cg10174836) had increased methylation levels in HCC. The three sites were defined as ACADS methylation site (MS)−1 (cg01535453), ACADS MS-2 (cg08618068), and ACADS MS-3 (cg10174836). The associations between alterations in DNA methylation levels at these sites and clinicopathological features of HCC were assessed. Only ACADS MS-2 was correlated with metastatic status (*P* < 0.05), pathological grade (*P* < 0.05), and TNM stage (*P* < 0.01) (Fig. 3E). Patients with high ACADS MS-2 methylation had shorter OS and TFS (Fig. 3F, *P* < 0.05).

Next, we examined the function of ACADS MS-2. Wild-type and mutated (MT) ACADS promoter luciferase plasmids were constructed and transfected into HCC cells. We observed a marked increase in luciferase activity in cells transfected with MT ACADS MS-2 (Fig. S2B). Mutation of MS-2 also increased *ACADS* mRNA levels in five HCC cell lines (Fig. 3G, *P < 0.05, **P < 0.01). Similarly, MT ACADS MS-2 significantly inhibited the proliferation, migration, and invasion of tumour cells (Fig. S2C–E). In summary, we concluded that ACADS MS-2 is the specific CpG island that is methylated in HCC and leads to decreased *ACADS* levels.

### Construction and validation of the prognostic ACADS nomogram

**Fig. 4:**
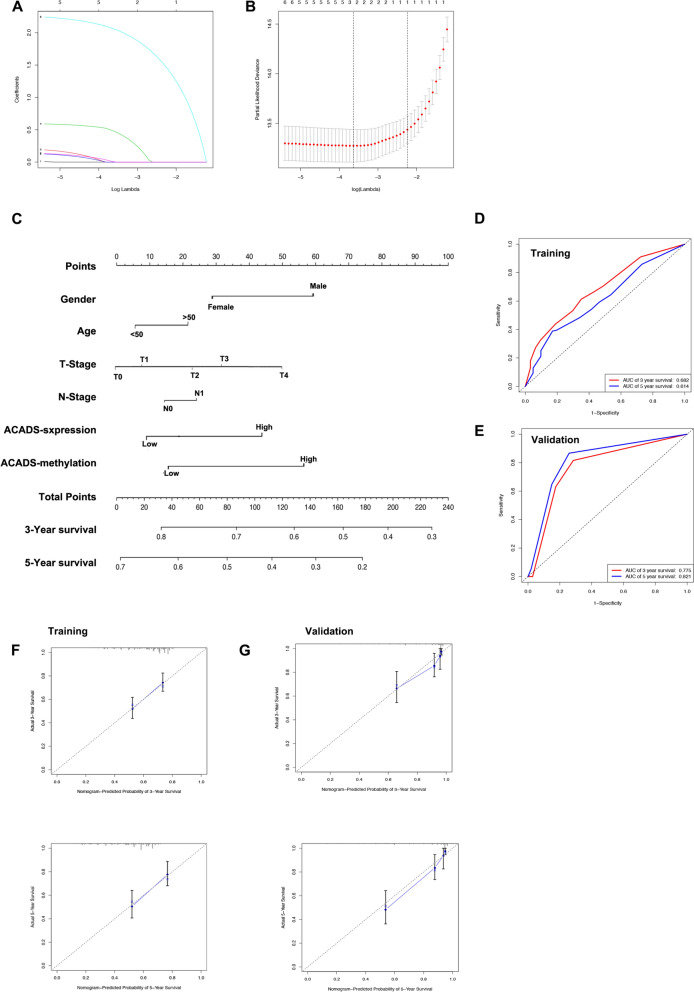
Construction of a novel nomogram based on ACADS methylation for HCC patients. **A** According to the tenfold cross-validation and the minimum criterion, the optimal penalisation coefficient lambda (λ) in the LASSO model was identified. **B** Six clinical features were identified using LASSO coefficient profiles. **C** The nomogram based on ACADS methylation in HCC. **D**, **E** ROC curve analysis was carried out to estimate the performance of the nomogram in predicting 3- and 5-year overall survival. **F** ROC curve analysis in the validation cohort. **G** Calibration curves comparing the predicted and actual survival

We enrolled 326 HCC patients from our centre and identified factors associated with prognosis using the LASSO algorithm. Sex, age, T stage, N stage, *ACADS* expression, and *ACADS* methylation were all associated with prognosis (Fig. 4A, B). A nomogram model including these factors was developed to estimate the 3- and 5-year OS rates of HCC patients who underwent tumour resection (Fig. 4C). The model had AUCs of 68.2% and 61.4% for 3- and 5-year survival, respectively, in the training cohort (Fig. 4D). In addition, TCGA data were used to validate the robustness of the nomogram. The AUCs were 77.5% and 82.1% for the 3- and 5-year OS, respectively, in the TCGA dataset (Fig. 4E). Calibration curves indicated good agreement between the observed OS and the predicted OS at 3 and 5 years in both the training and validation datasets (Fig. 4F, G; training dataset: 0.623 for 3-year survival and 0.657 for 5-year survival; validation dataset: 0.762 for 3-year survival and 0.724 for 5-year survival). Therefore, the nomogram has good predictive performance with respect to the estimation of OS and it can serve as a useful prognostic system for predicting the postoperative survival of patients with HCC.

### ACADS reprograms the tumour microenvironment in HCC

**Fig. 5:**
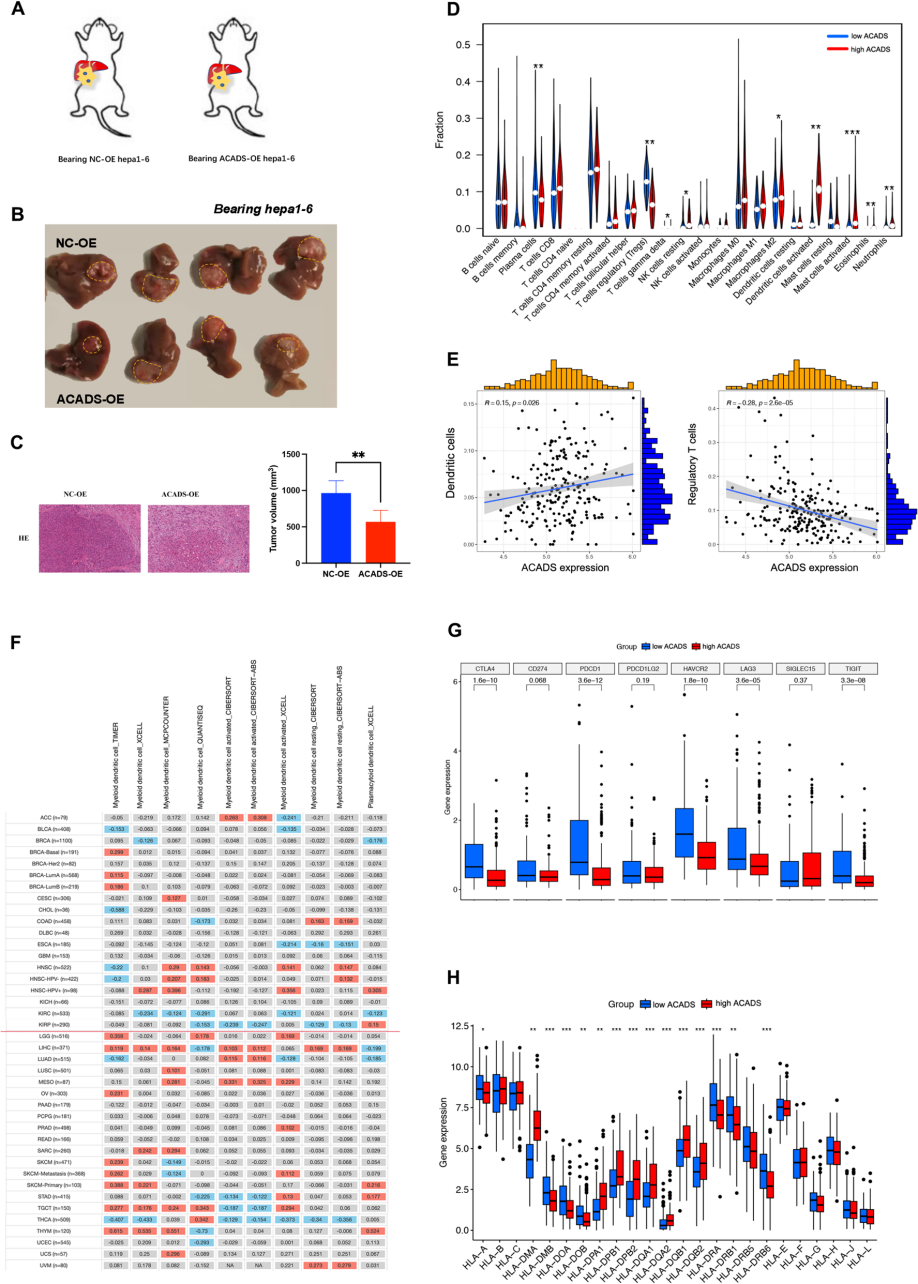
ACADS influences reprogramming of the TME in HCC. **A** Experimental design. **B** After the overexpression of ACADS in hepa1-6, the tumor burden got reduced in mice model. **C** Comparison of H&E staining analysis and tumor volumes between ACADS-OE Group and NC Group. **D** The distributions of various immune cells in the low- and high-ACADS cohorts in TCGA. The results were calculated using the CIBERSORT algorithm. **E** Pearson analysis indicated that ACADS overexpression could reduce infiltration of Tregs and enrich DCs in the HCC TME. **F** Heatmap of ACADS expression with infiltration of different immune cell types. **G**, **H** ACADS not only suppressed the expression of immune checkpoints but also increased the mRNA levels of HLAs (*P < 0.05, **P < 0.01, ***P < 0.0001)

**Fig. 6:**
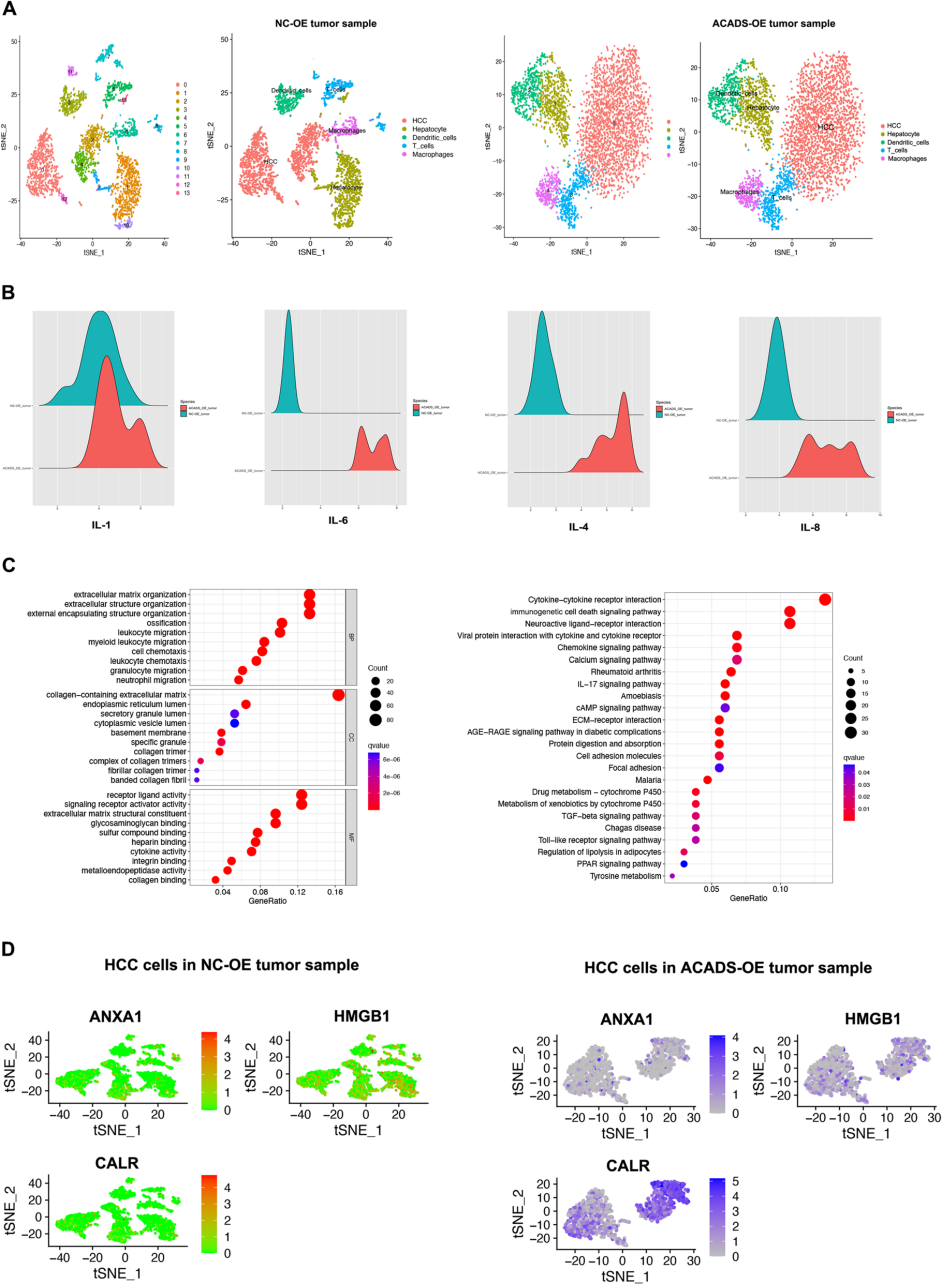
Immunogenic cell death could be mediated by ACADS in HCC. **A** scRNA-seq of tumour specimens from NC and ACADS-OE Hepa1-6 tumour-bearing mice. DCs were enriched in the microenvironment of ACADS-OE tumours. **B** ACADS enhances the secretion of DC effectors (IL-1, IL-4, IL-6, and IL-8). **C** GO and KEGG enrichment analyses of DEGs between NC and ACADS-OE HCC cells. **D** The expression patterns of AXNA1, HMGB1, and CALR using T-SNE analysis

Microenvironmental heterogeneity is a hallmark of cancer. Therefore, we created a mouse model of HCC using Hepa1-6 cells, a mouse hepatoma line, to explore the potential effects of ACADS on tumour microenvironment (TME) reprogramming (Fig. 5A). The tumour burden was lower in the ACADS-OE group than in the NC group (Fig. 5B, C, *P* < 0.05). And through HE staining experiments, we further found that ACADS overexpression inhibited the progression of HCC (Fig. 5C). Single-cell RNA sequencing (scRNA-seq) was then used to examine TME composition in the NC and ACADS-OE tumours. Dimension reduction analysis via the T-SNE method indicated that DCs were enriched in ACADS-OE tumours (Fig. 6A). We calculated the infiltration scores of immune cells in the TIMER database using the CIBERSORT algorithm, and the results showed that ACADS not only promoted the recruitment of DCs but also suppressed the infiltration of regulatory T cells in HCC (Fig. 5D, E). Similarly, there was a positive relationship between ACADS expression and DC infiltration in cancer (Fig. 5F). Encouraged by this finding, we analysed the variants of DC-associated effectors (including interleukin [IL]−1, IL-4, IL-6, and IL-8) in each group based on the scRNA-seq data mentioned above. Interestingly, increased mRNA levels of these effectors were observed in ACADS-OE tumours (Fig. 6B). We also evaluated the expression patterns of immune checkpoints and human leukocyte antigens (HLAs) in low- and high-ACADS HCC cohorts. As expected, the mRNA levels of most checkpoints were reduced in the high-ACADS cohort (Fig. 5G). The increase in ACADS also resulted in the reduced expression of HLAs (Fig. 5H). Therefore, ACADS could potentially enhance the antitumour response by accelerating DC activation in HCC.

### ACADS is associated with ICD in HCC

**Fig. 7:**
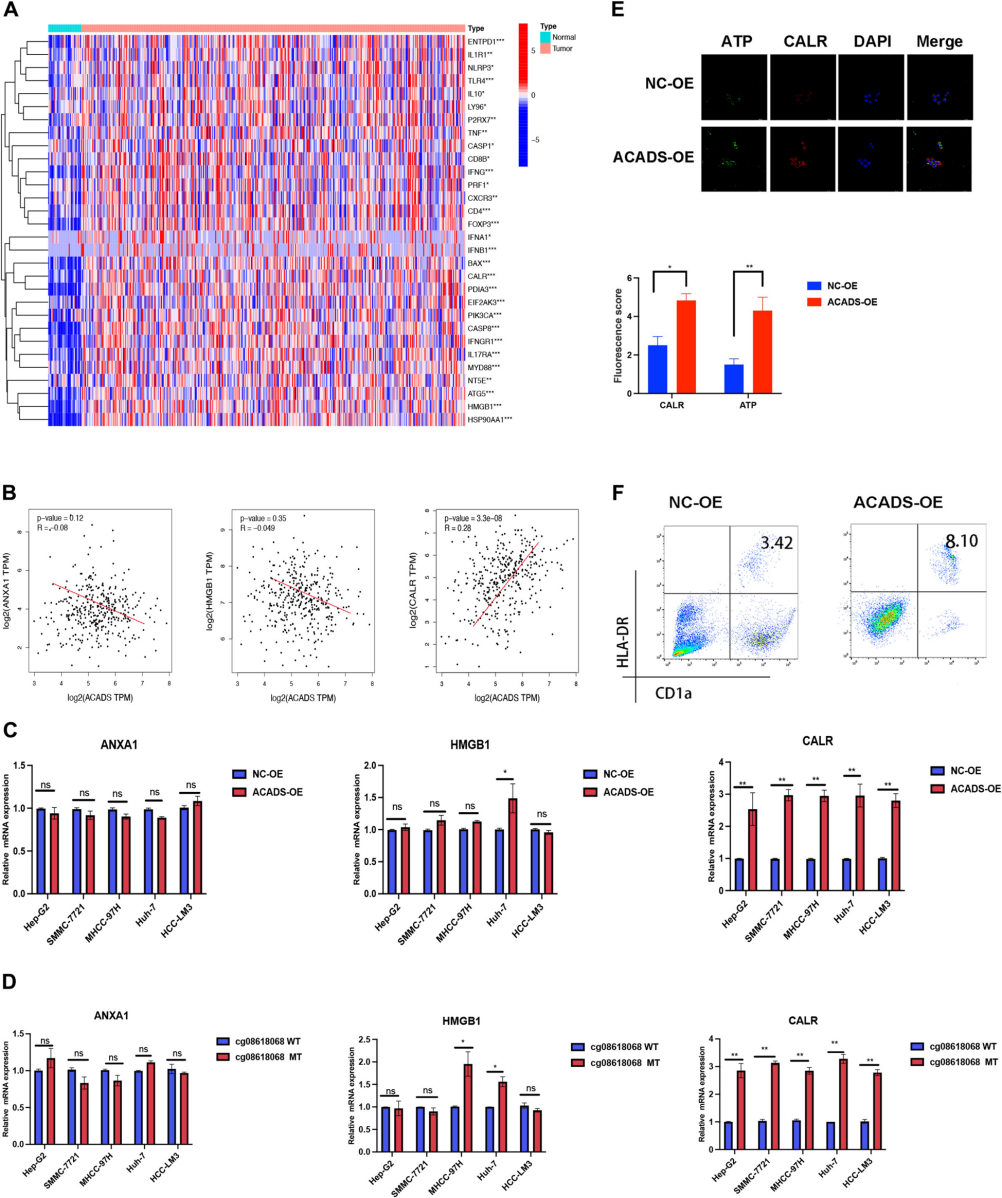
ACADS could promote ICD through upregulating CALR. **A** Compared to normal liver samples, *CALR* and *HMGB1* were upregulated in HCC tissues based on TCGA data. **B** Pearson’s correlation analysis of ACADS and CALR in HCC. **C** ACADS overexpression increased *CALR* mRNA levels in HCC cell lines as assessed by RT-qPCR. **D** Following mutation of ACADS MS-2, all tumour cells showed increased CALR expression. **E** Immunofluorescent staining assays of mice tumor tissues originated from NC or ACADS OE group were conducted. The up-regulation of ACADS resulted in the increasements of CALR protein and ATP (ICD marker) secretion in tumor. **F** Flow cytometry showed that ACADS overexpression accelerated the infiltration of DCs into the HCC microenvironment (*P < 0.05, **P < 0.01)

We identified differentially expressed genes (DEGs) between the high- and low-ACADs cohorts, and GSEA was performed (Fig. S3). The DEGs were enriched in immune cytokine receptor interactions. HCC transcriptional profiles were also isolated from scRNA-seq of NC or ACADS-OE mouse tumours. After identifying the DEGs in tumour cells from the different groups, we performed GO and KEGG enrichment analyses. As shown in Fig. 6C, ACADS influenced several immune-related signalling pathways, including ICD. ICD is a special type of cell death that maintains the activation of innate immunity and further enhances antitumour immune responses. High mobility group box 1 (HMGB1), AXNA1, and calreticulin (CALR) are important regulatory elements in ICD. Compared to normal liver samples, CALR and HMGB1 were overexpressed in HCC tissues (Fig. 7A). Combined with the tumour sequencing data obtained from HCC-bearing mice (Fig. 6D) and TCGA (Fig. 7B), these results suggest that ACADS specifically facilitates CALR expression. Subsequently, RT-qPCR was performed to verify whether CALR is regulated by ACADS. *CALR* mRNA increased following ACADS overexpression and mutation of ACADS MS-2 (Fig. 7C, D). Immunofluorescence assays were conducted on mouse tumour tissues originating from the NC and ACADS-OE groups. CALR and ATP (an ICD marker) secretion were both increased in ACADS-OE tumours (Fig. 7E, *P* < 0.05). Flow cytometry further indicated that ACADS overexpression accelerated the infiltration of DCs into the HCC microenvironment (Fig. 7F). Altogether, these results indicate that sustained enhancement of CALR derived from ACADS can trigger ICD in the TME of HCC.

## Discussion

To understand the biological contribution of ACADS as a tumour suppressor during pathogenesis, its expression and prognostic value were validated using the HCC cohort from TCGA. We also performed comprehensive in vitro and in vivo analyses, which showed that hypermethylation of ACADS MS-2 resulted in *ACADS* silencing and was correlated with pathologic grade, metastasis, and TNM classification in HCC. Our work also illustrates that ACADS facilitates the antitumour response by activating the CALR-mediated ICD.

Compared to normal liver cells, *ACADS* was decreased in all HCC cell lines. The Kaplan–Meier method showed that low *ACADS* expression was associated with poor OS and TFS in HCC patients. Consistent results were confirmed in tumour tissues using IHC.

Epigenetic alterations such as histone modification, DNA methylation, and miRNA-mediated processes are associated with various mechanisms of tumourigenesis in HCC. Some studies have indicated that *ACADS* can be epigenetically modified in various tumours [24–28]. The *ACADS* promoter was hypermethylated in the majority of solid tumours and negatively correlated with mRNA expression in a pan-cancer analysis, and we confirmed increased methylation of the *ACADS* promoter in HCC cells using BSP. In addition, we identified a potential CpG island site in the *ACADS* promoter, ACADS MS-2 (cg08618068), that was hypermethylated in HCC and associated with *ACADS* mRNA levels. ACADS MS-2 hypermethylation correlated with metastatic status, pathological grade, and TNM stage. Mutation of ACADS MS-2 was associated with decreased *ACADS* methylation and increased *ACADS* mRNA levels. Mutation of ACADS MS-2 also inhibited the proliferation, migration, and invasion of HCC cells. Therefore, MS-2 acts as a vital CpG island responsible for *ACADS* DNA methylation and HCC pathogenesis.

Based on these findings, we developed an ACADS-based nomogram. To the best of our knowledge, the model presented in this study is the first to apply ACADS expression as a methylation biomarker in a nomogram, and the nomogram had good predictive performance for predicting survival in HCC patients.

Interestingly, in an HCC mouse model using Hepa1-6 cells, high infiltration of DCs was observed in the ACADS-OE tumours. We suspect that ACADS enhances the innate immune response by facilitating ICD. Several studies have revealed that ICD is mainly mediated by damage-associated molecular patterns, including CALR, secreted ATP, and released HMGB1 [29]. Following ICD stimulation, immunogenicity increases, ultimately leading to an enhanced antitumour response. Using bioinformatics analysis and in vitro experiments, ACADS was confirmed to activate ICD by upregulating CALR in HCC. We also observed increased DCs recruitment in tumours with ACADS overexpression. Therefore, ACADS could be a potential immunotherapeutic target for HCC. However, no effective ACADS agonists have been identified so far. Therefore, there is an urgent need to develop novel drugs targeting ACADS.

Currently, research on ACADS as a drug target is evolving. In future studies, we intend to employ computational techniques to identify key active domains of ACADS, optimize the development strategy of ACADS agonists through fragment-based screening and covalent modification methods, and explore their potential role in drug development [30]. Furthermore, it would be beneficial to investigate whether analogues of existing metabolic regulators could modulate the activity of ACADS. We will also review the literature on related dehydrogenases that have been successfully targeted to gather insights that could be applied to ACADS. This exploration will be incorporated into our future research plans to enhance the translational potential of our findings. Additionally, regarding ACADS silencing, HCC cells may activate compensatory mechanisms or alternative metabolic pathways, which could undermine the efficacy of the agonists. Future research will also explore these mechanisms, particularly the compensatory roles of the glycolytic pathway and other short-chain dehydrogenases. For example, the fatty acid β-oxidation pathway, which is closely linked to ACADS function, may be redirected through alternative dehydrogenases such as medium- or long-chain acyl-CoA dehydrogenases (MCAD, LCAD) when ACADS is silenced [31]. Furthermore, alterations in mitochondrial function or lipid metabolism could contribute to metabolic reprogramming in HCC cells [32]. In future research, we plan to conduct pathway analysis and metabolic profiling to identify these compensatory mechanisms, and this could provide insights into combination therapies to overcome resistance.

## Conclusions

Our work clarified that ACADS is downregulated in HCC and its promoter is modified by DNA methylation. ACADS plays an essential role in activating ICD by upregulating CALR in tumour cells. A nomogram based on *ACADS* methylation was established to predict the prognosis of HCC patients. Therefore, ACADS may serve as a novel DNA methylation biomarker for HCC.

## Supplementary Information

Supplementary Material 1

Supplementary Material 2

Supplementary Material 3

## Data Availability

All relevant data can be found in the figures and supplementary materials. For any further details required to reanalyze the data presented in this current study, additional information can be obtained from the corresponding author upon reasonable request.

## Abbreviations

ACAD: Acyl-CoA dehydrogenase
ACADS: Acyl-CoA dehydrogenase short chain
DC: Dendritic cell
HCC: Hepatocellular carcinoma
HLA: Human leukocyte antigen
HMGB1: High mobility group box 1
ICD: Immunogenic cell death
IL: Interleukin
Me-DIP: Methylated DNA immunoprecipitation
BSP: Bisulfite sequencing PCR
scRNA-seq: Single-cell RNA sequencing
TFS: Tumour-free survival
TME: Tumour microenvironment
TSG: Tumour suppressor gene
