# Supplementary figures and images for: DNA methylation of ACADS promotes immunogenic cell death in hepatocellular carcinoma
Source: Cell Biosci. 2025 Jan 12;15:3. doi: 10.1186/s13578-024-01334-1 (PMC11727568; doi:10.1186/s13578-024-01334-1)

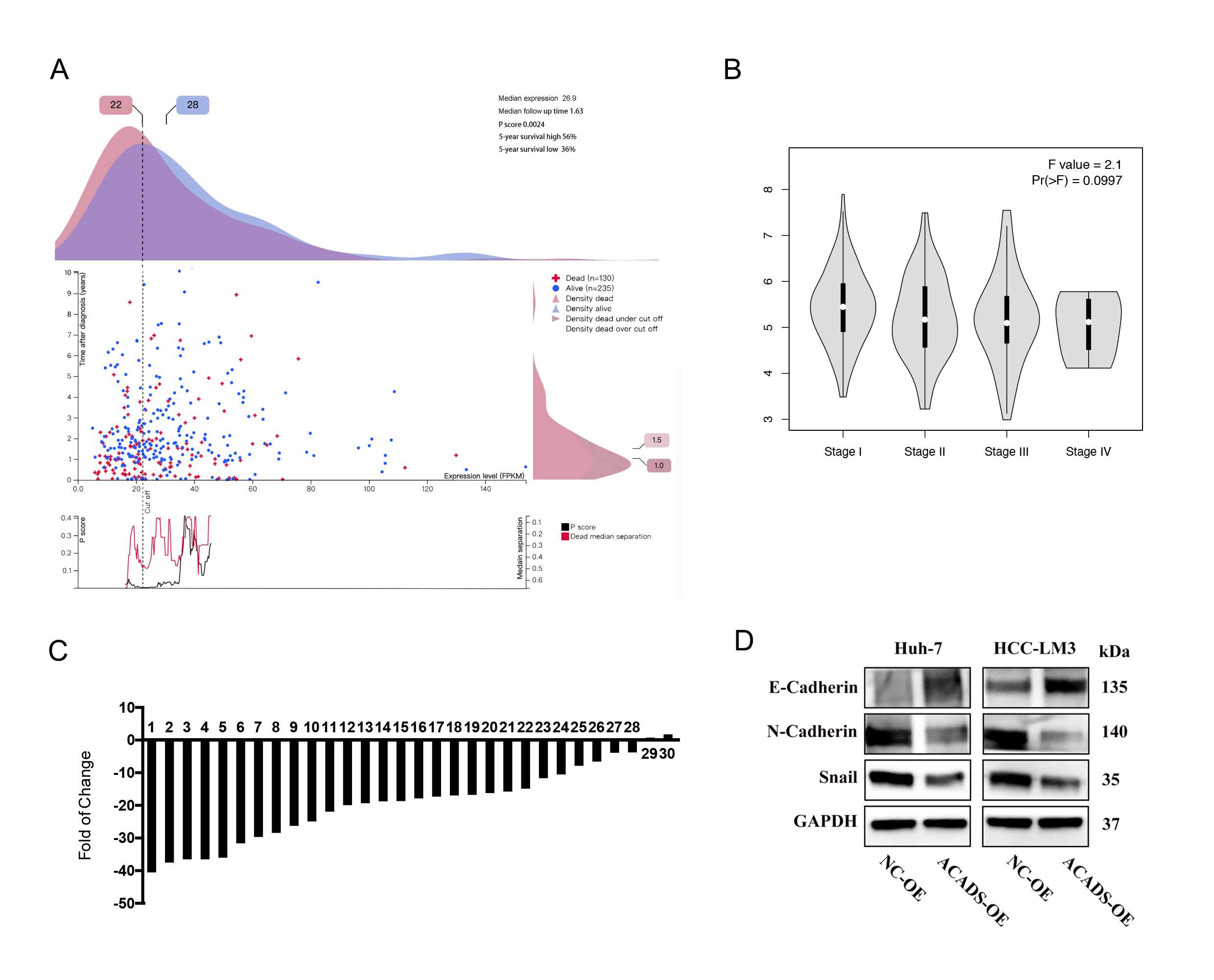

Supplement: Supplementary file 1 — Supplementary Material 1 [file 13578_2024_1334_MOESM1_ESM.tif]

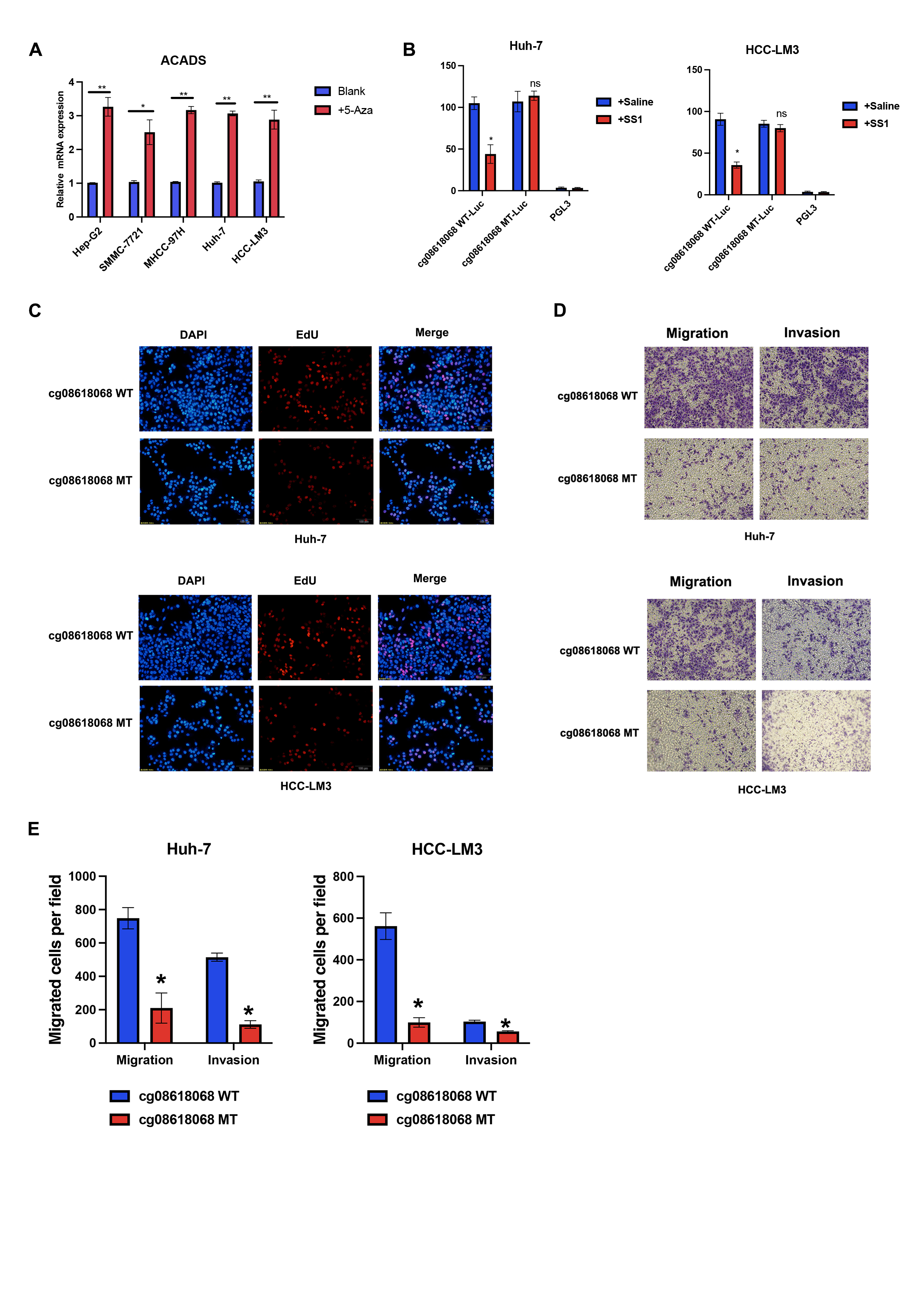

Supplement: Supplementary file 2 — Supplementary Material 2 [file 13578_2024_1334_MOESM2_ESM.tif]

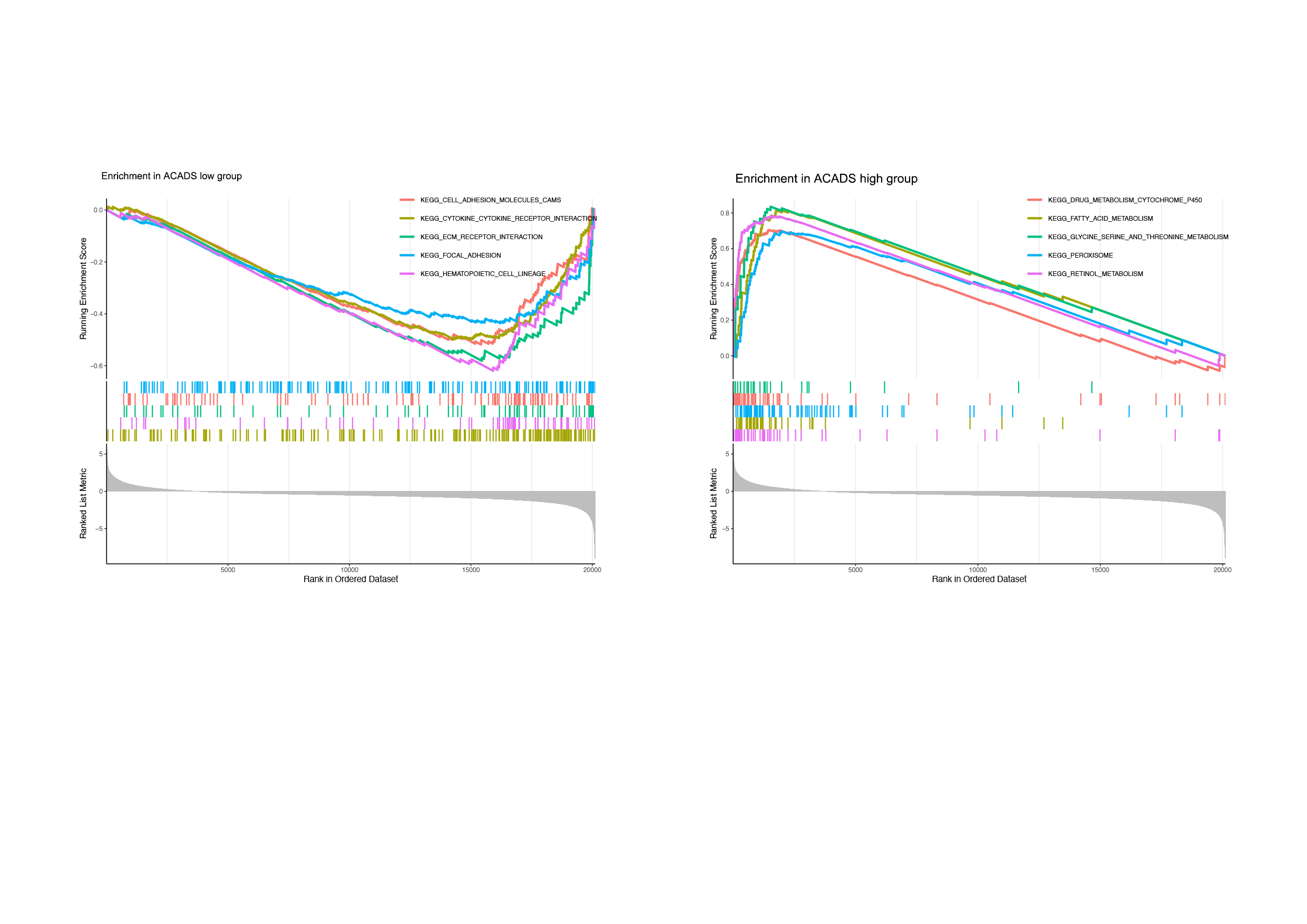

Supplement: Supplementary file 3 — Supplementary Material 3 [file 13578_2024_1334_MOESM3_ESM.tif]
